# Characterisation of GNSS Carrier Phase Data on a Moving Zero-Baseline in Urban and Aerial Navigation

**DOI:** 10.3390/s20144046

**Published:** 2020-07-21

**Authors:** Fabian Ruwisch, Ankit Jain, Steffen Schön

**Affiliations:** Institut für Erdmessung (IfE), Leibniz Universität Hannover, 30167 Hannover, Germany; schoen@ife.uni-hannover.de

**Keywords:** Global Navigation Satellite System (GNSS), kinematic terrestrial and flight experiment, geodetic and high sensitivity GNSS receivers, double difference, relative positioning, stochastic models

## Abstract

We present analyses of Global Navigation Satellite System (GNSS) carrier phase observations in multiple kinematic scenarios for different receiver types. Multi-GNSS observations are recorded on high sensitivity and geodetic-grade receivers operating on a moving zero-baseline by conducting terrestrial urban and aerial flight experiments. The captured data is post-processed; carrier phase residuals are computed using the double difference (DD) concept. The estimated noise levels of carrier phases are analysed with respect to different parameters. We find DD noise levels for L1 carrier phase observations in the range of 1.4–2 mm (GPS, Global Positioning System), 2.8–4.6 mm (GLONASS, Global Navigation Satellite System), and 1.5–1.7 mm (Galileo) for geodetic receiver pairs. The noise level for high sensitivity receivers is at least higher by a factor of 2. For satellites elevating above $30^{\circ }$, the dominant noise process is white phase noise. For the flight experiment, the elevation dependency of the noise is well described by the exponential model, while for the terrestrial urban experiment, multipath and diffraction effects overlay; hence no elevation dependency is found. For both experiments, a carrier-to-noise density ratio (C/N${}_{0}$) dependency for carrier phase DDs of GPS and Galileo is clearly visible with geodetic-grade receivers. In addition, C/N${}_{0}$ dependency is also visible for carrier phase DDs of GLONASS with geodetic-grade receivers for the terrestrial urban experiment.

## 1. Introduction

Accuracy, precision and availability requirements are very stringent in urban navigation, advanced driver assistance system (ADAS) and autonomous driving. In order to meet these requirements at a few cm to dm level, Global Navigation Satellite System (GNSS) carrier phase observations must be used in a relative positioning mode [1]; the observations and their uncertainty must be modelled adequately in the positioning filter. Unfortunately, a sound quality description of GNSS observations in kinematic applications with different receivers is still missing, which is important for a stringent quality control of the observations and subsequent improved integrity of the positioning application.

For static applications, double difference (DD) residuals of geodetic-grade receivers were tested in zero and short baseline setups, indicating optimum and more realistic performance including multipath, respectively. Also, the need for improvement in the Global Positioning System (GPS) stochastic models was stressed based on the obtained results, particularly elevation dependency of carrier phase observations per satellite and correlation between L1 and L2 phase observations [2,3]. Based on this type of study, adequate observation weighting models have been derived, for example, based on carrier-to-noise density ratios [4,5,6] or taking atmospheric correlations into account [7,8]. Specifically, in [4], the Sigma-ϵ model is proposed and its use in resolving the poor satellite geometry problem by considering data from low elevating satellites is described with a real data-set. The Sigma-ϵ model extended by the Danish method can be used to remove outliers due to multipath and diffraction from the phase data and consequently improve the positioning precision [5]. An improved observation weighting model based on signal-to-noise ratio measurements is proposed in [6]; it shows that about 10% more ambiguities could be resolved with low elevation data when compared to the standard elevation-dependent weighting model. A variance-covariance model (VCM) based on the turbulence theory is developed to explain the stochastic characteristics of carrier phase observations due to atmospheric fluctuations [7]. A study considering correlation among observations with a diagonal covariance matrix shows a significant improvement in the positioning solution of the simulated and real data-set compared with positions estimated using a standard diagonal dependent elevation model [8]. The performance of low-cost and geodetic-grade receiver combinations is studied by a static zero-baseline experiment [9]; results show that the DD carrier phase noise is about 2.2 mm with a low-cost receiver combination and convey the feasibility of integrating low-cost equipment in a multi-sensor system.

For characterisation of GNSS receivers in kinematic applications, position deviations or carrier phase residuals are analysed with respect to a reference trajectory often obtained from a GNSS/inertial measurement unit (IMU) tightly coupled solution [10,11]. Moreover, high sensitivity GNSS receivers can track very weak signals in comparison to geodetic-grade receivers [12]. It is also shown that high sensitivity GNSS receivers when fed with a miniaturised atomic clock would lead to an improvement in the precision of vertical position and velocity estimates. For real-time kinematic (RTK) processing over short baselines in an urban environment, multipath errors are significant and degrade the positioning results. In order to apply proper weighting to the observations, an elevation dependent exponential function weighting is proposed for GPS code range and phase range measurements [13]. It is shown that by using C/N${}_{0}$ weighting with appropriate models for phase observations, diffracted observations at high elevation could be identified [4,10]. A weighting model combining both elevation and C/N${}_{0}$ for both code and phase observations in open-sky and suburban scenarios for horizontal RTK positioning is explained in [14]. An innovation-based integrity monitoring scheme by using an Extended Kalman Filter (EKF) with the help of C/N${}_{0}$ weighting reduces the horizontal positioning error and protection level in an urban environment by 1.15 and 7.5 m, respectively [15]. Positioning errors computed with precise-point positioning (PPP) in a kinematic test drive for different mass market GNSS chipsets is less than about 0.35, 0.8 and 1 m in open sky, suburban and highway environments, respectively [16]. Positioning performance of several mass-market receivers along standardised road environments are explained in [17]. A RTK fixed solution is available at 50% of the time with a probability of continuity loss of 0.54 compared to 98% availability with a continuity loss probability of 0.045 for standard code phase positioning [18]. Different challenges with GNSS positioning in urban areas are explained; the performance improvement with multi-GNSS constellations has been demonstrated with both simulated and real data [19].

The change in the dynamics and environment in a kinematic scenario affects the functionality of GNSS receivers. In highly kinematic applications, the impact of improper receiver clock synchronisation leads to a bias in baseline estimation and also attitude determination. The attitude herein refers to the orientation of the object with regards to the local-level frame and typically comprises of the roll, pitch and yaw angles [20]. Different studies to compensate for the baseline bias and determine the attitude have been reported in [21,22]. In order to achieve high precision relative positioning in the case of extremely high velocity vehicles (e.g., spacecraft), a DD model is proposed in [23]; apart from the standard DD formulations, it includes additional terms considering the single difference range rate for each receiver. The dynamic performance of different GNSS receivers evaluated in open sky conditions with a drone (maximum operating velocity approximately 15 m/s) is explained in [24] and for acceleration determination with a 100 Hz data rate in [25].

In order to contribute to the GNSS carrier phase characterisation in kinematic applications, we present the performance analysis of carrier phase observations in kinematic terrestrial and aerial environments for high sensitivity and geodetic-grade GNSS receivers. Multiple receivers are configured together with an external clock, which has a high frequency stability. We can thus observe if there is any particular difference in the number of observations or estimated noise levels of the observations. The remainder of the paper is structured as follows. At first, the measurement configuration and experimental details of the urban test drive and flight test are explained in Section 2. In Section 3, the DD computation algorithm is described. Also, the importance of time synchronisation and consideration of user velocity while computing the DDs for relative positioning is highlighted. The analysis of the DD carrier phase observations with respect to different parameters is discussed in Section 4. Based on the analyses of DDs, stochastic models of carrier-phase observations are derived for kinematic terrestrial and aerial applications in relation to the different environments and receiver types. Finally, on the basis of the performance of different receiver combinations and different GNSS systems, conclusions are drawn in Section 5.

## 2. Experiment Description

Kinematic experiments are carried out using a vehicle and an aircraft to determine the quality of the carrier phase observations in different environments.

### 2.1. Terrestrial Experiment

#### 2.1.1. Measurement Configuration

**Figure 1:**
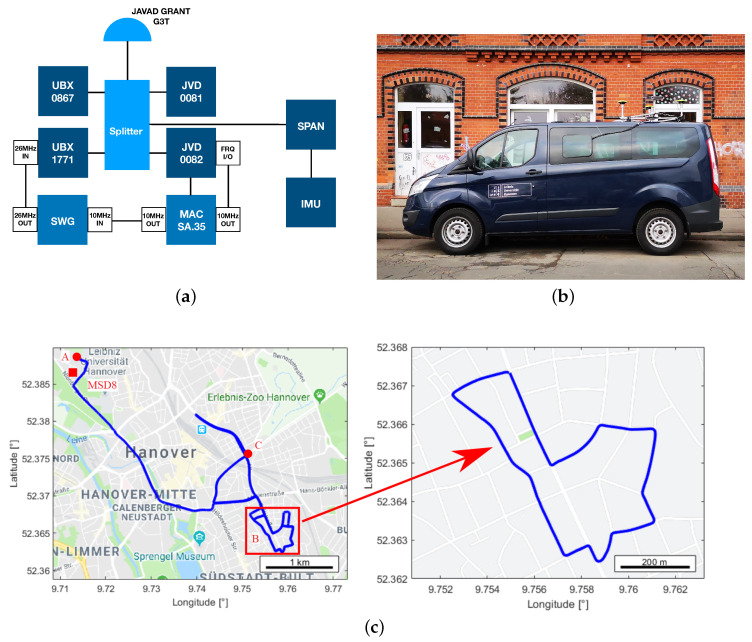
Kinematic experiment in Hannover. (**a**) Measurement setup: two JVD and two u-blox (UBX) receivers connected via a GNSS splitter to a geodetic antenna. One of each receiver types is connected to an atomic clock (MAC SA.35), SWG refers to signal wave generator and a Novatel SPAN receiver was logging inertial measurement unit (IMU) data. (**b**) Vehicle used during the experiment. (**c**) Measurement route. For details of the marked points, cf. Table 1. Map presentation taken from [26].

The geodetic antenna (JAVAD GrAnt G3T) on top of the IfE van is connected via GNSS splitter to two different receiver types, cf. Figure 1a,b. Two geodetic JAVAD (JVD) Delta TRE G3T receivers (JVD 0081, JVD 0082) together with two high sensitivity u-blox (UBX) NEO M8T receivers (UBX 0867, UBX 1771) form a zero-baseline on the roof of the vehicle. One of each receiver type is connected to an external Rubidium frequency standard (Microsemi MAC SA.35m). The signal wave generator serves as a link in-between the atomic clock and the UBX receiver to adapt the frequency [12]. The other two receivers were driven by their respective internal temperature controlled crystal oscillator (TCXO).

This zero-baseline configuration on a moving platform allows us to characterise the individual properties of geodetic and high sensitivity receivers and their combinations in an urban environment.

#### 2.1.2. Test Drive Details

The driven trajectory in Hannover (Figure 1c) represents typical characteristics of an urban area that are strong shadowing caused by buildings and variable velocities with standstill phases at traffic lights and crossings. The measurement starts with a standstill phase of approximately 10 min in a parking lot (label A). The test route led through streets with four-storey residential buildings of roughly 20 m height as well as through the inner city ring, which has a more open sky characteristic. The trajectory marked with the red box (label B) was repeatedly driven five times with approximately seven minutes per round. The details of the marked points on the urban trajectory map and their approximate GPS times are listed in Table 1. The total duration of the experiment is about two hours. Each receiver was recording GPS, GLONASS and Galileo code, phase and Doppler observations as well as signal strength with a sampling rate of 1 Hz. It must be noted that an elevation cut-off angle was not applied while recording the data. The geodetic-grade receivers have the capability to track multiple frequencies simultaneously while the high sensitivity receivers can only track the L1 frequency of each GNSS system. The receiver independent exchange format (RINEX) specifiers of the phase observation types recorded on single or multiple frequencies from different GNSS systems using different receiver types are listed in Table 2 [27]; corresponding code ranges, Doppler and signal strength were also recorded. Note that the frequency bands (L1, L2 and L5) for different GNSS systems are slightly different.

The reference trajectory was computed using data from the iMAR FSAS IMU and the GNSS reference station (referred to as MSD8) located at the rooftop of IfE. The ground-truth was determined in post-processing mode using the TerraPOS software [28].

### 2.2. Aerial Experiment

#### 2.2.1. Measurement Setup

**Figure 2:**
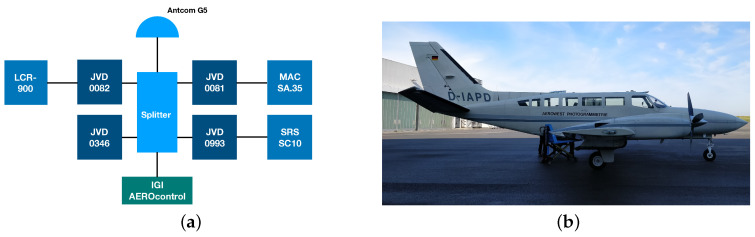
Flight experiment. (**a**) Measurement configuration, (**b**) aircraft used during the experiment.

The measurement setup and the aircraft used during the experiment is shown in Figure 2a,b, respectively. The measurement configuration consists of four geodetic-grade JVD GNSS receivers (type: Delta TRE-G3T(H)), which were running on the same firmware version. In addition to the four receivers, there were three external atomic clocks, IGI’s AEROcontrol unit and an active GNSS splitter, which was connected to the Antcom G5 antenna placed at the top of the aircraft fuselage.

The internal circuitry of three JVD Delta receivers (0081, 0082 and 0993) were driven by the three external oscillators (Microsemi MAC SA.35m, Spectratime LCR 900 and SRS SC10), respectively. On the contrary, the fourth JVD Delta receiver (0346) was driven by its internal TCXO. The IGI’s AEROcontrol is a navigational grade IMU integrated with a high precision Septentrio GNSS receiver. From the data captured with the IGI’s AEROcontrol unit, a precise kinematic reference trajectory was computed for the complete duration of the flight experiment.

**Figure 3:**
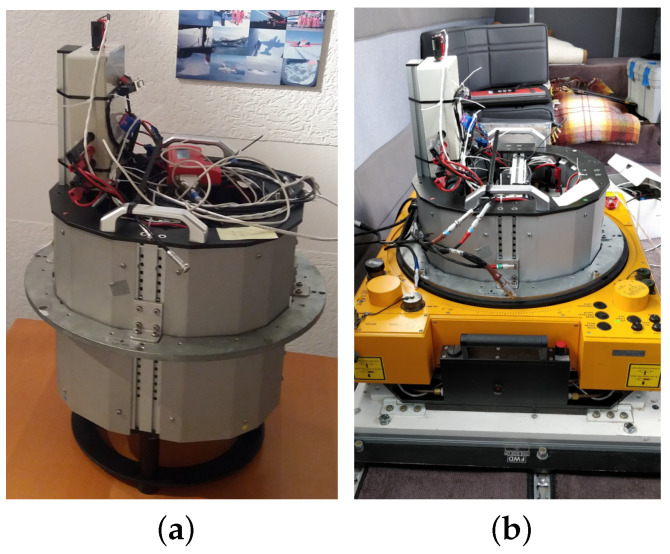
(**a**) Measurement devices placed within a camera sensor pod. (**b**) Photogrammetery mount within the aircraft on which the sensor pod is fixed.

The whole measurement setup except for the GNSS antenna was fixed within a camera sensor pod, as shown in Figure 3a. Later, this sensor pod was placed inside the aircraft on a passively dampened aerial photogrammetry mount, as depicted in Figure 3b. The passive dampening on the mount reduces the impact of sudden jerks, mechanical shocks and vibrations on the external oscillators and prevents them from permanent damage.

#### 2.2.2. Flight Test Details

The flight experiment was carried out on 7th October 2019 around the surrounding area of Dortmund airport in Germany using a modified twin-engine Cessna 404 TITAN aircraft (Figure 2b). The experiment lasted for a duration of about three hours. GPS, GLONASS and Galileo pseudorange, carrier phase and Doppler measurements were recorded on all the receivers, except JVD 0346, which recorded only GPS and GLONASS observations. The recorded observation RINEX specifiers on L1, L2 and L5 frequencies for different GNSS systems were exactly the same as stated for the geodetic receiver in Table 2.

**Figure 4:**
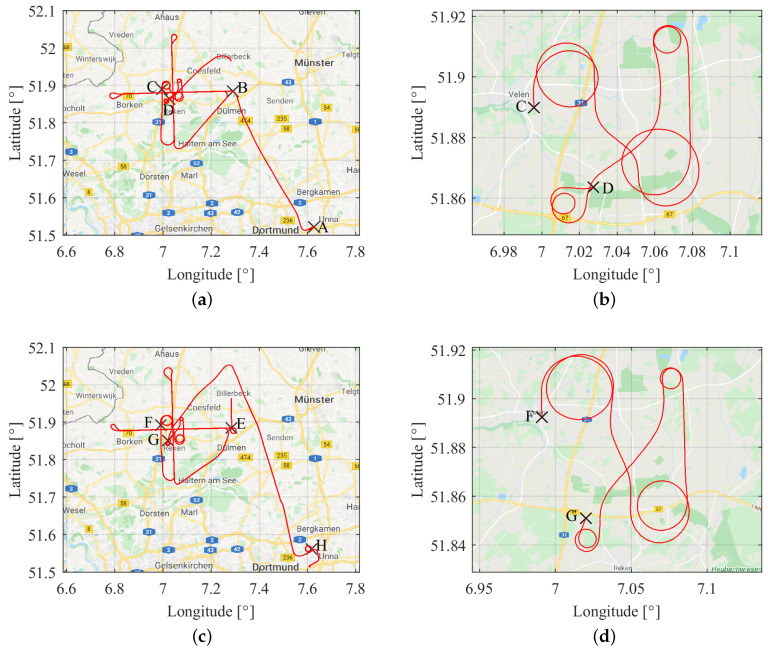
Airborne trajectory at heights of about 650 and 2800 m in (**a**) and (**c**), respectively. Dynamic manoeuvre segments corresponding to the different heights in (**b**) and (**d**), respectively. For details of the marked points, cf. Table 3. Map presentations taken from [26].

The sampling rate was set to 10 Hz for all the receivers. In the course of the experiment, an identical set of flight manoeuvres were performed at two different ellipsoidal heights, particularly at a lower height level of about 650 m and at an upper height level of about 2800 m. A single set of these manoeuvres comprised of a straight and level flight followed by flight turns with varying roll angles. The first straight and level flight starts moving from east to west and then back to east, followed by a straight and level flight line again from south to north and then back to south. After these flight paths, flight turns with high dynamics were conducted. At first, two flight turns with maximum roll angles of up to ± 25${}^{\circ }$ were performed, followed by two flight turns with maximum roll angles up to ± 58${}^{\circ }$. It must be noted that higher the roll angle, the steeper the flight turn. All the flight manoeuvre operations carried out at a lower altitude (≈ 650 m) are referred to as phase 1 of the flight experiment. A similar set of manoeuvres were conducted at an upper altitude (≈ 2800 m); it is referred to as phase 2 of the flight experiment. Figure 4a,c show the complete flight trajectory path in phase 1 and phase 2, respectively. There are four distinct points marked with different labels on each flight path in phase 1 and phase 2. The information of all the marked points on both the trajectories are summarised in Table 3 along with the corresponding approximate GPS time. Moreover, flight turns which involved high dynamics in phase 1 and phase 2 are depicted in Figure 4b,d, respectively.

**Figure 5:**
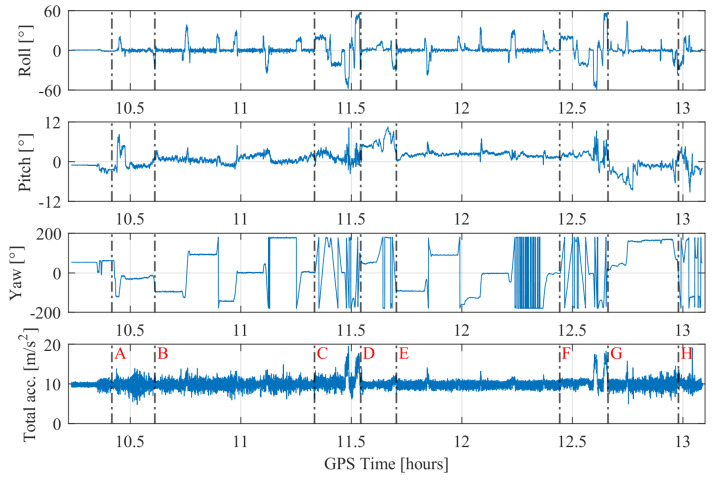
Flight dynamics recorded by the IMU along the whole trajectory. The black dashed line indicates different marked points, which are explained in Table 3. Marked point labels are only inserted in the last subplot but apply to all time series.

The flight dynamics (attitude and total acceleration) recorded by the IGI’s IMU are shown in Figure 5. The black dashed lines in Figure 5 corresponds to the different marked points in the flight trajectories (cf., Figure 4). The flight dynamics include roll angles up to ± 59${}^{\circ }$ and pitch angles in the range of ± 10${}^{\circ }$. The flight dynamics are the highest towards the end of both the dynamic manoeuvres (i.e., point D and G) where the flight is accelerating at magnitudes greater than 2 g and turning with the highest roll angles. The phases where the pitch angles are seen increasing steadily refer to the take-off phase and flight ascend phase to a higher altitude. On the contrary, the phases in which pitch angles are seen decreasing steadily refer to flight descend phase to a lower altitude and the landing phase. The phases wherein the yaw angles are constant refer to straight and level flight phases; the dynamics during these phases are not very high.

### 2.3. Pre-Processing of the GNSS Data-Sets

The recorded observations were collected in a receiver-specific binary format, which was later converted to the RINEX version 3.03 [27]. In the case of JVD receivers, the JVD proprietary JPS2RIN software [29] was used to convert the binary data into RINEX format. In order to convert the UBX binaries, the *convbin* tool of the RTKLib software was used [30]. It supports the conversion of the proprietary UBX multi GNSS raw observations (binary RAWX format).

In order to pre-process the observations, the IfE-GNSS-Toolbox was utilised. It was implemented and maintained by the working group Positioning and Navigation (Prof. Schön) at the IfE. In post-processing, the calculated reference trajectories were used to compute epoch-wise corrections. These correction values are subtracted from the collected raw data (code ranges, carrier phases and Doppler), which leads to the so-called Observed-Minus-Computed (OMC) values that can also be called pre-fit residuals. Unless explicitly stated otherwise, the following results always refer to the OMC values.

## 3. Double Difference Observation Modelling

### 3.1. Double Difference Principle

Combining carrier phase measurements from two stations or receivers *A* and *B* and two satellites *i* and *k*, DD can be formed, which eliminates common error sources and largely reduces similar propagation specific effects:

(1) $$\begin{array}{cc} \Phi_{AB}^{ik} & =\Phi_{A}^{i}-\Phi_{A}^{k}-\left(\Phi_{B}^{i}-\Phi_{B}^{k}\right) \end{array}$$

(2) $$\begin{array}{cc} \; & =\lambda N_{AB}^{ik}+\epsilon_{AB}^{ik} \end{array}$$

where Φ is the carrier phase measurement. In a perfectly synchronised zero-baseline configuration, the only remaining part in $\Phi_{AB}^{ik}$, is the DD integer ambiguity $N_{AB}^{ik}$ and a noise term $\epsilon_{AB}^{ik}$ that contains the carrier phase noise and also remaining multipath effects if, for example, different types of receivers are used in the processing. Hence, DD is an efficient way of analysing the characteristics and the noise of the carrier phase observations.

### 3.2. Time Synchronisation

To compute DD, observations from two different receivers are combined. Each receiver is driven by a different oscillator that realises a different time scale [31]. Depending on the technology and processing philosophy of the manufacturer, these time scales can be steered internally to a GPS time scale at different accuracy levels (e.g., μs or even better). Sometimes, the oscillators are completely free running or with an internal adjustment to GPS time within ± 0.5 ms. When the clock error reaches this threshold, a 1 ms jump is introduced. A special case occurs for the high sensitivity UBX receivers: the time tag of the raw observations is not an integer second but can be an arbitrary time offset in the range of milliseconds. The impact of an asynchronism between the receiver clocks needs to be considered in relative positioning techniques as stated in the different texts, for example, [32] or [33]. The bias due to poor time synchronisation can be resolved by using extrapolation [22] or by aligning the receiver time scales [21,34].

For kinematic cases, the asynchronism between the receiver clocks induces additional baseline components depending on the dynamics of the platform. For our applications and encountered receiver clock offsets, the following linear correction is sufficient:

(3) $$\begin{array}{c} b_{AB}^{ik}={\left(s_{B}^{i}\left(t\right)-s_{B}^{k}\left(t\right)\right)}^{⊺}\cdot v\left(t\right)\cdot \left(t_{A}-t_{B}\right) \end{array}$$

where $s_{B}^{i}\left(t\right)$ and $s_{B}^{k}\left(t\right)$ denote the line of sight unit vectors between the platform and the satellites i and k at the nominal reception time t, respectively, v(t) is the velocity of the platform at that time and ⊺ represents the transpose operator. The correction term depends further on the relation of the actual reception times of the receivers $t_{A}$ and $t_{B}$ that includes the receiver clock biases. When applying the correction to the computed DD, the kinematic DD bias needs to be subtracted:

(4) $$\begin{array}{cc} {\tilde{\Phi }}_{AB}^{ik} & =\Phi_{AB}^{ik}-b_{AB}^{ik} \end{array}$$

**Figure 6:**
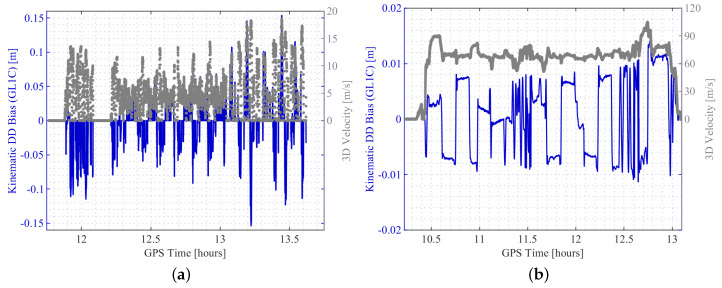
Kinematic double difference (DD) bias. (**a**) Terrestrial experiment: Kinematic DD bias of GPS L1 signal exemplarily shown for receiver combination UBX 0867–UBX 1771 and satellite combination PRN 29–PRN 26 (**b**). Aerial experiment: Kinematic DD bias of GPS L1 signal exemplarily shown for receiver combination JVD 0081–JVD 0082 and satellite combination PRN 3–PRN 1.

In Figure 6, two examples of the computed kinematic DD bias are shown, one for each experiment. For the urban scenario, the kinematic DD bias is computed for the UBX receiver combination 0867-1771 and GPS satellite pair PRN 29-26. For the flight experiment, the kinematic DD bias is computed for the geodetic-grade receiver combination 0081-0082 and GPS satellite pair PRN 3-1. The magnitude of the correction term highly depends on the velocity of the moving platform and the relative receiver clock biases. Hence, the correction values are almost zero in stand still phases of the urban area test drive. In epochs where the velocity is about 15 m/s, the correction values increase to more than about 10 cm. The kinematic DD bias values for the high sensitivity equipment are quite high due to the large time offsets as stated earlier.

The magnitude of correction during the flight experiment is lower even though the aircraft is moving at speeds of more than 100 m/s during some phases. This is specifically due to the smaller relative clock biases of the two JVD receivers, which were connected to external atomic clocks. However, changes in the values still follow a behaviour, which can be explained by the dynamic manoeuvres and consequential changes of the heading and the change in the speed of the aircraft.

These results clarify that the computation of the kinematic DD bias correction term is essential for precise relative positioning.

### 3.3. DD Computation Algorithm

**Figure 7:**
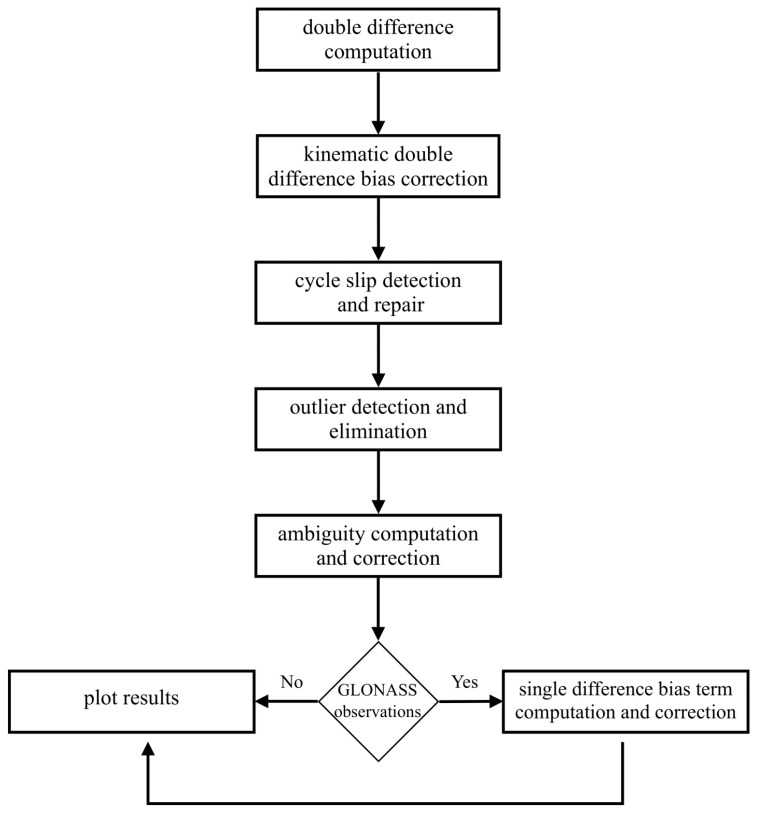
Double difference processing procedure.

To analyse the quality of carrier phase observations using DDs, additional processing steps are required. All the steps for the computation of DD from OMC, to the presentation of the results are depicted in the flow chart in Figure 7.

After computing the DDs (cf. Equation (2)) the kinematic DD bias correction term is subtracted from the DD to obtain ${\tilde{\Phi }}_{AB}^{ik}$ (cf. Equation (4)). Carrier phase observables are affected by cycle slips, which mainly occur due to low signal strength, signal losses and re-acquisition of the signal by the receiver [35,36]. In this algorithm, cycle slips are detected and subsequently repaired using triple differences. In order to obtain a true indication on the statistical behaviour of the signals, an outlier detection is applied based on a threshold detection method. Epochs wherein the triple difference is exceeding the threshold of 1.2 cm (i.e., the 3-σ value of the optimal carrier phase noise [36]) are deleted and hence, outliers are eliminated. Further, the DD integer ambiguity term needs to be computed and subtracted for each satellite arc. Due to the zero moving baseline, the integer ambiguity can be determined by just rounding to the nearest integer and is expressed as:

(5) $$\begin{array}{c} N_{AB}^{ik}=\mathrm{round}\left(\frac{\mathrm{med}\left(\Phi_{AB}^{ik}\right)}{\lambda }\right) \end{array}$$

where $\mathrm{med}\left(\Phi_{AB}^{ik}\right)$ is the median of the DD of one continuous arc and λ the wavelength of the signal.

After subtraction of DD integer ambiguities, the GPS and Galileo DD can be used to assess the noise behaviour. Since GLONASS distinguishes satellites based on frequency-division multiple access (FDMA) technology, each satellite has a unique frequency. This leads to an additional bias referred to as single difference bias term when combining observations from two different satellites [37,38]. This bias is considered in the computation algorithm.

By applying all the steps as explained in the flowchart, DDs are obtained which have approximately a zero-mean for the complete time series. Statistics related to the computation of DD for two receiver combinations from each experiment (terrestrial and aerial) are summarised in Table 4. The larger number of ambiguities set up in the terrestrial experiment corresponds to more data gaps underlining that the signal reception conditions have a big impact on the quality of the observations. This can be seen clearly in the statistics as the number of cycle slips, outliers and ambiguities are higher whereas the number of observations is lower in the case of urban environments. The Galileo L1 signal seems more stable in the urban setup. In the case of the flight experiment, the count of cycle slips, outliers and ambiguities are considerably larger for receiver combination JVD 0993-0346 compared to JVD 0081-0082. On the contrary, the number of observations is almost similar for both the combinations.

## 4. Results Analysis

### 4.1. Urban Test Case

The measurement configuration offers the possibility to compare the signal quality of geodetic-grade and high sensitivity equipment. Therefore, two combinations (JVD 0081–JVD 0082 and UBX 0867–UBX 1771) are utilised to compute DD based on the strategy developed in Section 3. These two configurations are selected to point out major differences in the noise levels of the observed carrier phases for different GNSS systems. Note that the corrected DDs of all satellites are superimposed per system in each figure. Since the UBX receivers are only capable of tracking one frequency per system, the C/A code based carrier phase observations on the L1 frequency are analysed.

**Figure 8:**
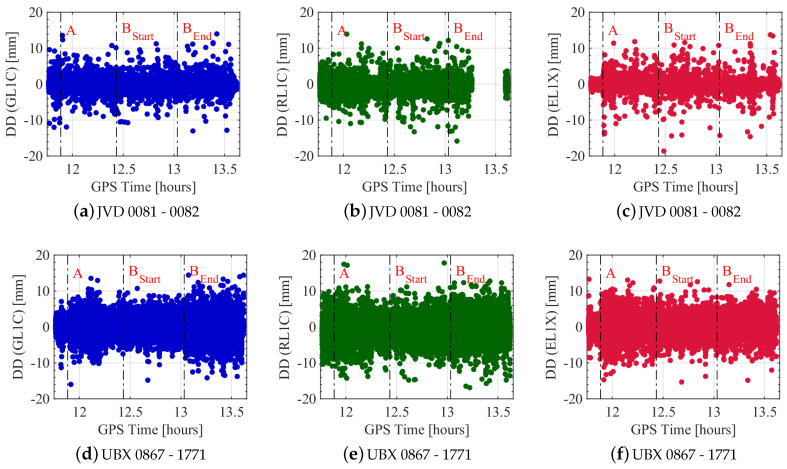
DDs of L1 carrier phase observations from all satellites for different receiver combinations with respect to GPS time. GPS, GLONASS and Galileo DD are shown in blue, green and red colours, respectively. Black dashed vertical lines indicate different points of the trajectory, which are explained in Table 1.

Figure 8 shows the L1 carrier phase noise of GPS, GLONASS and Galileo with respect to GPS time. The DDs of the geodetic-grade combination are depicted in the upper row while the DDs of the high sensitivity combination are depicted in the lower row. The DD noise of the different systems does not differ very much and vary roughly between ± 2 mm (Figure 8a,c). However, there are many outliers of up to 15 mm, which affects the time series. The same applies to the DDs of the UBX receivers. The major difference is the general magnitude of noise, which is more than doubled to roughly ± 5 mm.

The data gap in the GLONASS time series in Figure 8b results from the single difference bias computation (cf. Section 3). The integer DD ambiguity can be estimated if the bias term is smaller than 0.1 cycles. In order to keep the bias term smaller than 0.1 cycles, the single difference ambiguity must be known with an accuracy of 285 cycles for the minimum wavelength difference between two satellites. Similarly, this ambiguity must be known with an accuracy of 12 cycles for the maximum wavelength difference between two satellites [37]. This requirement for determining the single difference bias term is not met at these epochs and hence, a further analysis of the DD is not feasible. This is the reason why they are simply deleted.

Additionally noticeable is that fewer outliers appear for GPS and Galileo DDs in the stand still phase of the vehicle, which is the time until the first vertical line labelled with A. Also, the noise is lower in the static phase of the experiment. However, this does not apply to GLONASS DDs. During the repeatedly driven five rounds of the trajectory between labels B${}_{\mathrm{Start}}$ and B${}_{\mathrm{End}}$, neither a change in the noise level nor in the appearance of outliers is visible.

**Figure 9:**
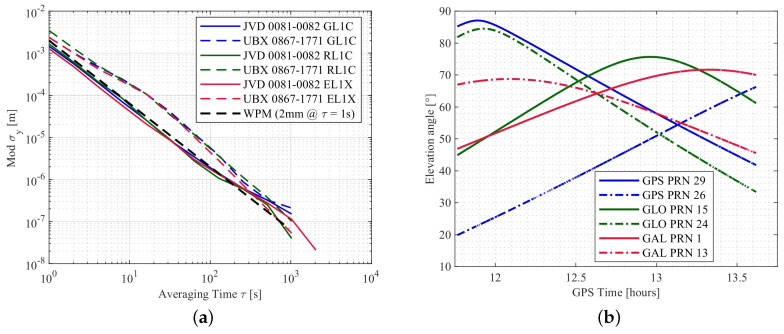
Modified Allan deviations of the L1 carrier phase DDs for one satellite pair of each GNSS system with two different receiver combinations in (**a**). For GL1C, RL1C and EL1X, DDs of PRN 29–26, PRN 15–24 and PRN 1–13 are used, respectively. The elevation angles of these satellites are shown in (**b**).

The noise characteristics of the DD time series are analysed using the Allan deviation (ADEV), which is the square root of the Allan variance [39,40]. To be more precise, the modified ADEV $\mathrm{mod}\;\sigma_{y}\left(\tau \right)$ is computed as it can distinguish between white phase noise and flicker phase noise. Since the ADEV is computed for continuous time series and this data set includes many data gaps due to signal interruptions, the DD time series from one satellite pair per system, which is almost continuous, is selected for this analysis. The modified ADEV’s are depicted in Figure 9a. The corresponding elevation angles for these satellite pairs with respect to GPS time is shown in Figure 9b. The noise level can be obtained at τ=1 [s]. The modified ADEV supports the statement that the noise level of the DD time series of the UBX combination is higher compared to the JVD combination; this applies to all the systems. At τ=1 [s], the values for the DDs of the geodetic combination is even below 2 mm. The slope of the respective curves is an indicator for the underlying noise process. For all combinations and signals, a white noise process for the carrier phase DDs with a slope of $\tau^{-3/2}$ is indicated in the figure for at least the first 100 s.

**Figure 10:**
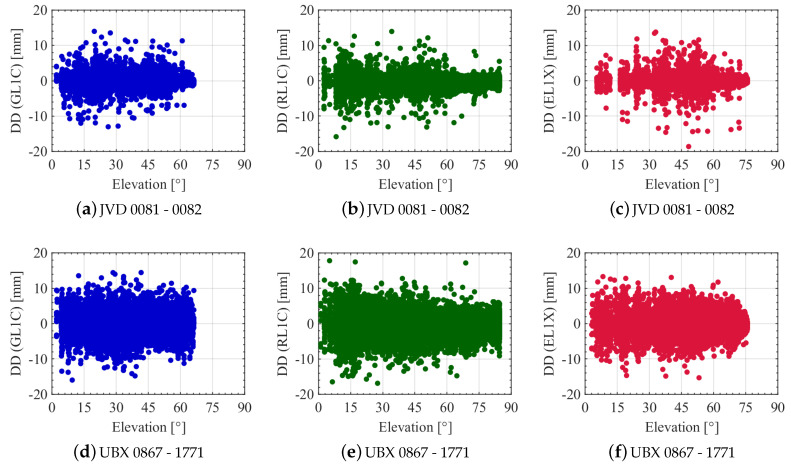
DDs of L1 carrier phase observations from all satellites for different receiver combinations with respect to the elevation angle of the non-reference satellites. GPS, GLONASS and Galileo noise levels are shown in blue, green and red colours, respectively.

In order to carry out the stochastic analyses of the DDs, the computed DDs are investigated with respect to the elevation angles of the non-reference satellites (cf., Figure 10). A clear elevation dependency, which can be expected at least in static experiments, is not visible in any of the combinations and GNSS systems. Especially, the Galileo DDs of the JVD receivers show properties of even higher noise and more outliers for elevations between 30${}^{\circ }$ and 60${}^{\circ }$. This is due to the fact that this range of elevations is particularly critical. It is shown in [41,42] that most of the signal reflections occur in these elevation ranges due to the building heights in the immediate vicinity of the antenna.

**Figure 11:**
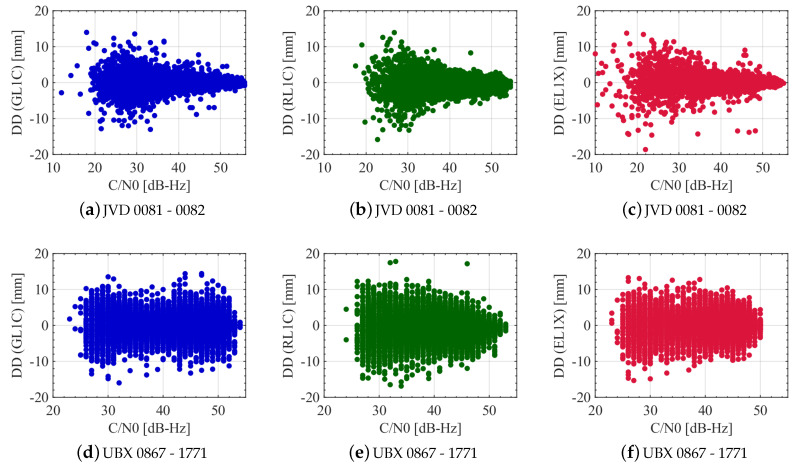
DDs of L1 carrier phase observations from all satellites for different receiver combinations with respect to the C/N${}_{0}$ values of the non-reference satellites. GPS, GLONASS and Galileo noise levels are shown in blue, green and red colours, respectively.

Since the magnitude of the DD noise is not particularly elevation dependent, the C/N${}_{0}$ is considered as another measure to characterise the observation quality. The results are depicted in Figure 11. Note that the x-scaling is different for the different receiver combinations. The behaviour of the DD noise with respect to the C/N${}_{0}$ values of the non-reference satellites highly differs considering one receiver combination with the other. For the geodetic-grade combination (cf. Figure 11a–c) there is a high C/N${}_{0}$ dependency visible. The DD noise is increasing for low C/N${}_{0}$s and is decreasing when the C/N${}_{0}$s are increasing. This applies similarly for all three GNSS systems. Note that due to receiver-internal settings, the recording of observations is stopped when the C/N${}_{0}$ is roughly less than 20 dB-Hz. The behaviour of the DD noise of the UBX receivers with respect to C/N${}_{0}$ is shown in Figure 11d–f. Note that the resolution of the C/N${}_{0}$ is only 1 dB-Hz. A clear C/N${}_{0}$ dependency is hardly visible. The receivers are well-known as high sensitivity equipment as it has the capability of tracking more signals even if the ray is blocked from buildings. Looking at Table 5, this is underlined for code ranges on all L1 frequencies. The number of recorded code observations is always higher compared to the geodetic-grade receiver. Investigating the carrier phase observations on the same frequencies, the numbers reveal an opposite behaviour in most cases.

Depending on the GNSS system analysed, the UBX receivers are recording 17% to 26% less carrier phase than code observations while the JVD receivers are recording the exact same number of code and phase observations. The computed DDs are in the C/N${}_{0}$ range of 25 to 54 dB-Hz, which leads to the assumption that the analysed UBX receivers are not capable of continuously tracking carrier phase observations in challenging situations and low C/N${}_{0}$ scenarios.

**Figure 12:**
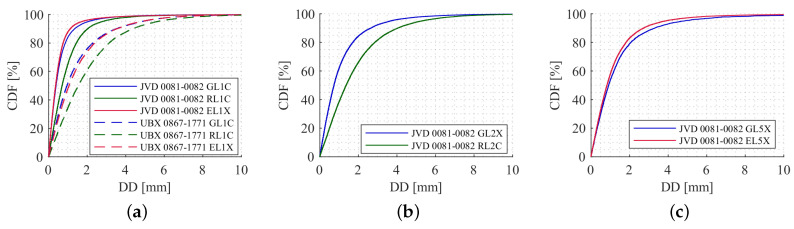
Cumulative distribution functions (CDF) for carrier phase DDs from all satellites on L1 (**a**), L2 (**b**) and L5 (**c**) frequencies for GPS (blue), GLONASS (green) and Galileo (red). The geodetic-grade combination is displayed with solid lines, the high sensitivity combination with dashed lines.

Figure 12 depicts the cumulative distribution functions (CDFs) for carrier phase DDs on different frequencies. Investigating the JVD DDs, 95% of the values for GPS, GLONASS and Galileo are below 2, 2.8 and 1.7 mm, respectively. For the UBX DDs, 95% of the values for GPS, GLONASS and Galileo are below 4.8, 5.6 and 4.7 mm, respectively. To get an idea of the quality of the carrier phases on the other frequencies, the CDFs of L2 frequencies and L5 frequencies are shown in Figure 12b,c. This is only feasible to analyse with the JVD combination, since the UBX receivers were tracking only one frequency. Compared to the L1 results, the quality of L2 and L5 DDs are relatively poor. For GPS and GLONASS L2 DD, 95% of the values are less than about 3.6 and 5.3 mm, respectively. For GPS and Galileo L5 DD, 95% of the values are below 4.8 and 3.8 mm, respectively. These results underline that the quality of GPS and Galileo carrier phase observations are similar, whereas the quality of GLONASS carrier phase observations is worse in comparison.

### 4.2. Flight Test Case

All the following analyses concerning the flight experiment were carried out using two receiver combinations operating in zero-baseline mode. The first combination is between JVD receivers 0081-0082, both of which are connected through external atomic clocks while the second combination is between JVD receivers 0993-0346, wherein receiver 0346 is driven through its internal TCXO. These two combinations were selected to observe any particular changes in the noise levels due to different clock configurations. Also, DD estimates from all the satellites in a constellation are superimposed in each subplot.

**Figure 13:**
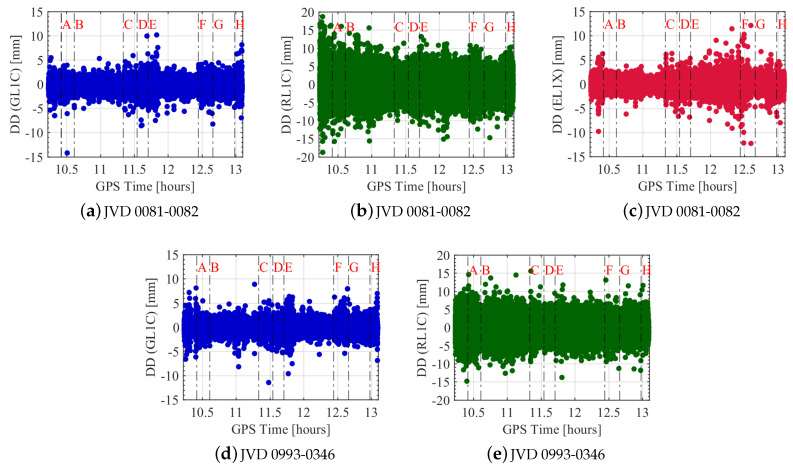
DDs of L1 carrier phase observations from all satellites for different receiver combinations with respect to the flight experiment time. GPS, GLONASS and Galileo noise levels are shown in blue, green and red colours, respectively. Black dashed vertical lines indicate different flight phases, which are explained in Table 3.

Figure 13 depicts the L1 carrier phase DDs of the moving zero-baseline for GPS, GLONASS and Galileo with respect to GPS time. They are computed as explained in Section 3. The GNSS system specific (GPS and GLONASS) noise dispersion are almost similar for both the receiver combinations, except that the number of outliers for the first receiver combination is higher for GLONASS observations compared to the second receiver combination. This is mainly due to the large number of outliers deleted for the second receiver combination during the DD estimation process compared to the first (cf. Table 4). Apart from a few outliers, the deviation of GPS L1 carrier phase is about 2 mm for the complete flight trajectory. In the case of the GLONASS L1 carrier phase, the noise is slightly higher during the start of the flight experiment (segment: start-A) compared to the other flight segments of the first receiver combination. During the segment (start-A), the aircraft was static for about 6 min and then was moving at a low velocity towards the runway. Along the complete trajectory, the sudden noisy spikes seen correspond to the flight turn, which resulted in a change of flight roll and heading angles. Overall, the deviation for GLONASS is about 5 mm for both receiver combinations, which is 2.5 times higher compared to GPS. For the first receiver combination, the spread of Galileo L1 carrier phase noise is about 2 mm for the complete flight trajectory, similar to GPS. It is also seen that the values of Galileo L1 phase noises are slightly higher at the upper altitude (segment E-G) compared to those at a lower altitude (segment A-D). Finally, there are no GNSS system specific significant changes that are seen in the computed noise levels during the highly dynamic manoeuvres (segments C-D, F-G).

**Figure 14:**
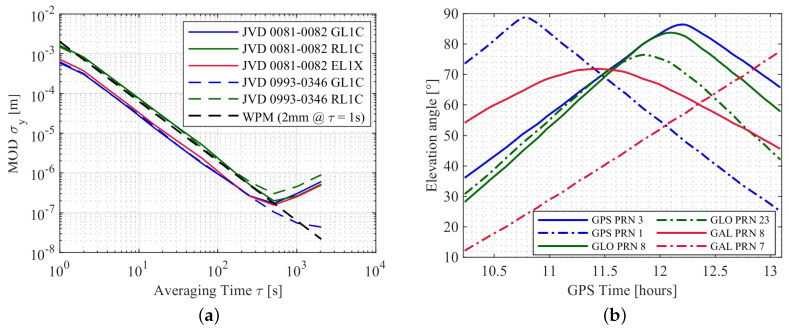
Modified ADEV’s of the L1 carrier phase DDs for one satellite pair of each GNSS system with two different receiver combinations in (**a**), corresponding elevation angles of the satellites in (**b**). Solid and dash-dot lines represent reference and the other satellite in (**b**), respectively.

Similar to the urban case, the underlying process noise of the carrier phase observations are analysed with the modified ADEV’s. The estimated DDs from one satellite pair of each GNSS system and two different receiver combinations are used to compute the modified ADEV’s and are shown in Figure 14a. The corresponding elevation angles of the different satellites are depicted in Figure 14b. It is seen that the noise process of L1 carrier phase observations from different systems resembles largely white phase noise (WPM) for up to τ=500 [s]. Moreover, receiver combinations with different clock configurations does not have a large impact on the quality of the observations. Also, the higher noise level of the GLONASS L1 observation is clearly visible compared to GPS and Galileo L1.

**Figure 15:**
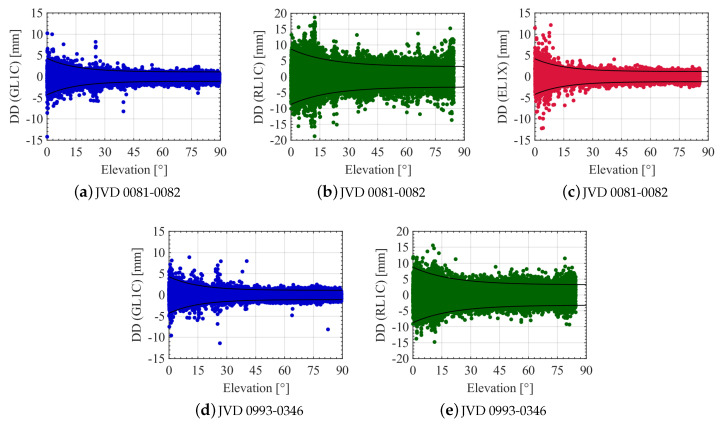
DDs of L1 carrier phase observations for different receiver combinations with respect to the satellites elevation angles. GPS, GLONASS and Galileo noise levels are shown in blue, green and red colours respectively. Solid black curves represent exponential functions that fit to the estimated DDs.

The stochastic behaviour of GNSS L1 carrier phase DDs is analysed in connection with satellites elevation angles and the received C/N${}_{0}$ of the corresponding signals. Figure 15 shows the DDs for GPS, GLONASS and Galileo L1 carrier phases with regards to the satellite elevation angles. All the sudden spikes observed in computed GNSS DD at elevation angles greater than about 10${}^{\circ }$ are due to recorded data from an ascending satellite or just before the loss of visibility of a satellite already in view. An elevation dependency is observed for all the computed DDs. The standard deviation of the phase range dependent on the elevation angle *h* can be approximated using the following exponential function [13]:

(6) $$\begin{array}{c} \sigma \left(h\right)=a_{0}+a_{1}\cdot \exp\left(\frac{-h}{h_{0}}\right) \end{array}$$

where $a_{0}$, $a_{1}$ are constant terms; $h_{0}$ represents the scaling factor for the elevation angle. The values listed in Table 6 are derived empirically to model all the estimated L1 carrier phase system specific DDs with all the receiver combinations. As examples, decreasing exponential functions for two different receiver combinations evaluated using the parameters in Table 6 can be seen in all plots of Figure 15. The corresponding increasing exponential function seen in Figure 15 is equivalent to a change in the sign of decreasing exponential function. From Figure 15, an elevation dependent weighting scheme modelled as an exponential function is justified for aerial navigation applications requiring higher accuracy and precision.

**Figure 16:**
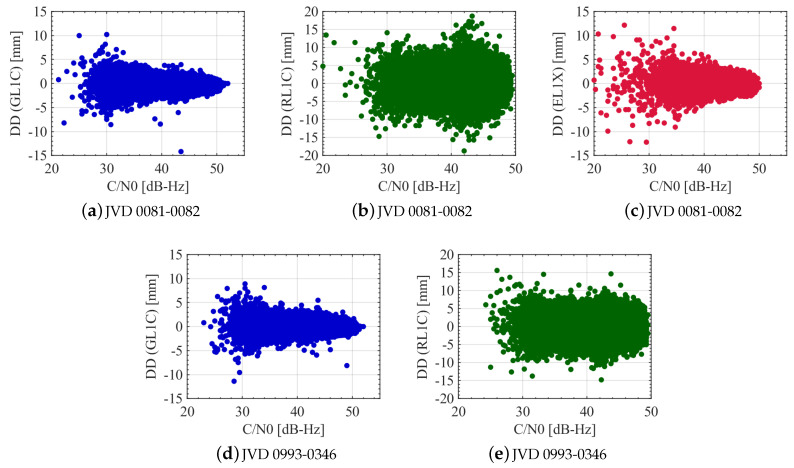
DDs of L1 carrier phase observations for different receiver combinations with respect to the received C/N${}_{0}$ from all the satellites. GPS, GLONASS and Galileo noise levels are shown in blue, green and red colours, respectively.

The DDs for GPS, GLONASS and Galileo L1 carrier phases in relation to the received signal’s C/N${}_{0}$ from different satellites are shown in Figure 16. It is observed that the behaviour of GPS L1 DDs is similar for both the receiver combinations. In the case of GLONASS, the DDs varies largely randomly with the C/N${}_{0}$ and no correlation is observed for both receiver combinations. A C/N${}_{0}$ dependency is visible for GPS and Galileo, wherein large C/N${}_{0}$ leads to smaller noise levels in most cases and vice versa. As explained in the elevation dependency scenario, few outliers seen at large C/N${}_{0}$ are due to the addition of a new visible satellite or loss of visibility of a satellite during the experimental campaign. Based on the analysis, a C/N${}_{0}$ dependent weighting scheme is also suitable for GPS and Galileo L1 phase observations in aerial applications.

**Figure 17:**
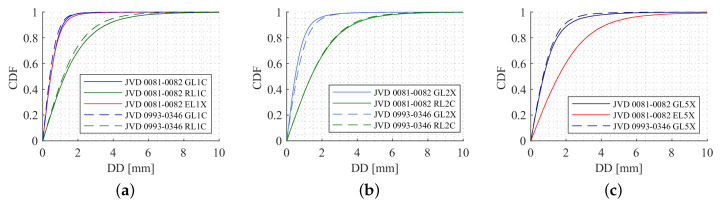
DDs CDFs for L1 (**a**), L2 (**b**) and L5 (**c**) carrier phase observations from all the satellites of different systems with two different receiver combinations.

In order to see the noise behaviour of L2 and L5 carrier phase observations alongside L1, DDs are computed and analysed using CDFs. Figure 17a–c shows the CDF for L1, L2 and L5 carrier phase observations, respectively. For both the receiver combinations, 95% of the DD values for GPS, GLONASS and Galileo L1 are less than about 1.4, 4.6 and 1.5 mm, respectively. Similarly, for GPS and GLONASS L2, 95% of the DD values are less than about 2 and 4.7 mm, respectively. Finally, for the GPS and Galileo L5 observations, 95% of the DD estimates are less than about 2.8 and 5.4 mm, respectively. For other receiver combinations, the noise levels are almost similar with regards to L1, L2 and L5 carrier phase observations. Even though the noise level is slightly higher for both L1 and L2 GLONASS DDs, the values are acceptable in the case of kinematic data processing. There is a significant increase in the noise level of Galileo L5 observation when compared to its L1 data-set.

## 5. Conclusions

A detailed analysis of GNSS carrier phase DDs in urban and flight navigation is presented. Two experiments were conducted, one using a vehicle and another using an aircraft to study the quality of the carrier phase observations. During the flight experiment, high dynamics are affecting the geodetic-grade equipment. In contrast to the open sky characteristics, peculiar in-flight navigation, the experiment carried out in the urban area was affected by obstructions and diffraction from multiple buildings and the environment was more prone to severe multipath effects. In addition to the geodetic-grade receivers, the urban experiment also included high sensitivity receivers.

In order to characterise the quality of the phase observations, carrier phase DDs are analysed on a moving zero-baseline. In both the experiments, the time synchronisation of receivers needs to be considered to account for the kinematic DD bias. It is shown that not taking the kinematic DD bias into account leads to systematic errors in the range of several centimeters depending on the receiver clock biases and the velocity of the moving platform.

In the urban environment, it is observed that the noise of DDs for UBX receivers (high sensitivity) is higher in comparison to the geodetic receivers. In the case of geodetic-grade receiver combination, 95% of the L1 carrier phase noises for GPS, GLONASS and Galileo are below 2, 2.8 and 1.7 mm, respectively. These values are at least two times higher with the UBX receivers. For all the systems, the noise process of the DD time series for high elevating satellites is dominated by a white phase modulation as indicated by the modified ADEV. An elevation dependency is not observed due to multipath contamination from buildings in the vicinity of the antenna. On the contrary, a C/N${}_{0}$ dependency is observed and a corresponding weighting can be applied for geodetic-grade receivers. Interestingly, the availability of the UBX carrier phase data seems restricted compared to the geodetic receivers.

There is no impact of high dynamics on the analysed DDs with geodetic-grade receivers with the data from the flight experiment. Also, there is no significant difference observed between the two selected receiver combinations, which means that different ultra-stable external oscillator configurations do not have any influence on the quality of the observations. For both receiver combinations, 95% of the L1 carrier phase noises for GPS, GLONASS and Galileo are below 1.4, 4.6 and 1.5 mm, respectively. All DD time series show a clear white noise process, similar to the urban case. The DDs show a clear dependency with respect to the elevation angles and the C/N${}_{0}$ (except GLONASS DD with respect to C/N${}_{0}$). An elevation dependency based on an exponential function with different parameters for L1 carrier phase observations of different GNSS systems have been evaluated and listed for geodetic-grade receivers, applicable in an open-sky environment.

## Figures and Tables

**Table 1:** Details of marked points on the urban trajectory map.

Label Points	Urban Experiment Details	Approximate GPS Time
A	Start of the experiment. Standstill phase.	11:45:00
A	End of the standstill phase.	11:53:00
B (B${}_{\mathrm{Start}}$)	Repeatedly driven trajectory. Five rounds in total.	12:26:00
B (B${}_{\mathrm{End}}$)	End of the repeatedly driven trajectory.	13:02:00
C	End of the experiment.	13:37:00

**Table 2:** Receiver independent exchange format (RINEX) specifiers of recorded phase observations on different frequencies with geodetic and high sensitivity receivers.

GNSS System	Geodetic Receiver	High Sensitivity Receiver
GPS	L1C, L1W, L2X, L2W, L5X	L1C
GLONASS	L1C, L1P, L2C, L2P	L1C
Galileo	L1X, L5X	L1X

**Table 3:** Details of marked points on the flight trajectory maps.

Label Points	Flight Experiment Details	Approximate GPS Time
A	Flight take off phase beginning	10:25:00
B	Start of straight and level flight phase at the lower altitude, particularly flight beginning to move from east to west	10:36:40
C	Beginning of dynamic manoeuvres (first two flight turns with roll angles up to ± 25${}^{\circ }$ and later two steep flight turns with roll angles up to ± 58${}^{\circ }$) at lower altitude	11:20:00
D	Flight starts ascending to a higher altitude	11:32:30
E	Start of straight and level flight phase at a higher altitude, flight again starts moving from east to west	11:42:15
F	Beginning of dynamic manoeuvres at a higher altitude (first two turns with roll angles of up to ± 25${}^{\circ }$ and later again two steep turns with roll angles up to ± 59${}^{\circ }$)	12:26:30
G	Flight starts descending phase	12:39:40
H	Beginning of the flight landing phase	12:58:45

**Table 4:** GNSS L1 DD computation statistics for different receiver combinations from both experiments.

Rec. Pair	# Observations			# Cycle Slips			# Outliers			# Ambiguities		
	GPS	GLO	GAL	GPS	GLO	GAL	GPS	GLO	GAL	GPS	GLO	GAL
JVD (urban) 0081-0082	38,157	35,387	27,517	1632	1154	569	851	794	189	3318	3028	582
UBX (urban) 0867-1771	34,805	33,399	27,783	723	1064	80	407	632	116	3082	3249	1160
JVD (aerial) 0081-0082	136,285	87,464	75,594	82	68	40	59	71	43	426	419	174
JVD (aerial) 0993-0346	136,167	87,660	-	342	193	-	180	192	-	750	676	-

**Table 5:** Number of L1 code range (C) and carrier phase (L) observations of three satellite systems of two geodetic-grade receivers (JVD 0081 and 0082) and two high sensitivity receivers (UBX 0867 and 1771) during the test drive.

	GPS L1			GLONASS L1			Galileo L1		
	C	L	Ratio [%]	C	L	Ratio [%]	C	L	Ratio [%]
JVD 0081	39,558	39,558	0	37,144	37,144	0	28,201	28,201	0
JVD 0082	39,795	39,795	0	36,647	36,647	0	28,177	28,177	0
UBX 0867	46,405	35,368	–24	45,301	33,940	–25	33,884	28,034	–17
UBX 1771	48,402	36,201	–25	46,028	33,986	–26	34,475	28,111	–18

**Table 6:** Empirical values of the exponential function parameters—JVD Delta receivers.

Observation Type	a${}_{0}$ [mm]	a${}_{1}$ [mm]	h${}_{0}$ [${}^{\circ }$]
GL1C	1.1	3.2	15
RL1C	3.2	5.5	20
EL1X	1.2	3	15
